# Prevalence, associated factors and prevention of burnout in psychiatry trainees in Central and North West London NHS Foundation Trust

**DOI:** 10.1192/bjo.2021.206

**Published:** 2021-06-18

**Authors:** Grace Xia, Ahrane Jayakumar

**Affiliations:** Central and North West London NHS Trust

## Abstract

**Aims:**

To assess burnout, resilience, professional quality of life and coping mechanisms in Central and North West London psychiatry trainees

**Objectives:**

To determine Key factors associated with stress and burnout in workplace Effects of burnout on patient care and doctors Coping mechanisms used by trainees

**Background:**

Burnout is a well established condition that has been recently reported to affect a third of doctors. Psychiatrists in particular represents a high risk group among doctors for experiencing burnout, alcohol and drug use, posing suicide risk and other forms of work related stress.

**Method:**

The study comprised of a cross sectional questionnaire survey which included measure of stress (General Health Questionnaire), burnout (Maslach Burnout Inventory), and satisfaction with medicine as a career and personality (Big Five). During October to December 2019, core trainee and specialty trainee doctors in CNWL were asked to complete an online survey via emails.

**Result:**

We collected data from 50 CNWL psychiatry trainees. The sample consisted of 20 females (40%) and 30 males (60%). Ages varied from 26–58 years old, with a median age of 28. Core trainees (CT1–3) were recorded as 72% and specialty trainees at 28%.

Of those who responded, around half of the trainees (52%) experienced high levels of stress outside of work in their personal life. The most common causes that trainees felt makes psychiatry a stressful profession were violence and fear of violence, limited resources, dealing with confrontational patients, inability to affect systemic change and increasing culture of blame. Around half of respondents (54%) felt that they have experienced burnout but only 26% of respondents knew where to go to find resources to help cope with burnout. Physical exercise and speaking to colleagues were the most common coping mechanisms used by trainees to deal with stress.

Free text responses on what can be improved in workplace to enhance a positive experience of work included improving multidisciplinary interactions, easily accessible resources and increasing staffing levels. 74% of respondents felt they continued to care about what happens to patients regardless of working conditions.

**Conclusion:**

Half of CNWL trainee doctors who responded have experienced burnout. Some factors associated with stress and burnout in doctors are unique to psychiatry profession. Free text responses were useful in identifying areas for improvement in work places and useful coping mechanisms, which can be used to inform prevention and implement interventions to tackle burnout.

